# Supplementary material for: Deciphering Conformational Changes of the GDP-Bound NRAS Induced by Mutations G13D, Q61R, and C118S through Gaussian Accelerated Molecular Dynamic Simulations
Source: Molecules. 2022 Aug 30;27(17):5596. doi: 10.3390/molecules27175596 (PMC9457619; doi:10.3390/molecules27175596)
Supplement: Supplementary file 1 [file molecules-27-05596-s001.zip › molecules-1864686-supplementary.pdf]

molecules

MDPI

*Supplementary Materials*

# Deciphering Conformational Changes of the GDP-Bound NRAS Induced by Mutations G13D, Q61R, and C118S through Gaussian Accelerated Molecular Dynamic Simulations

**Zhiping Yu [1], Hongyi Su [2], Jianzhong Chen [3] and Guodong Hu [1,2,*]**

[1] Shandong Key Laboratory of Biophysics, Institute of Biophysics, Dezhou University, Dezhou 253023, China
[2] Laoling People's Hospital, Dezhou 253023, China
[3] School of Science, Shandong Jiaotong University, Jinan 250357, China
* Correspondence: xzszhgd@163.com

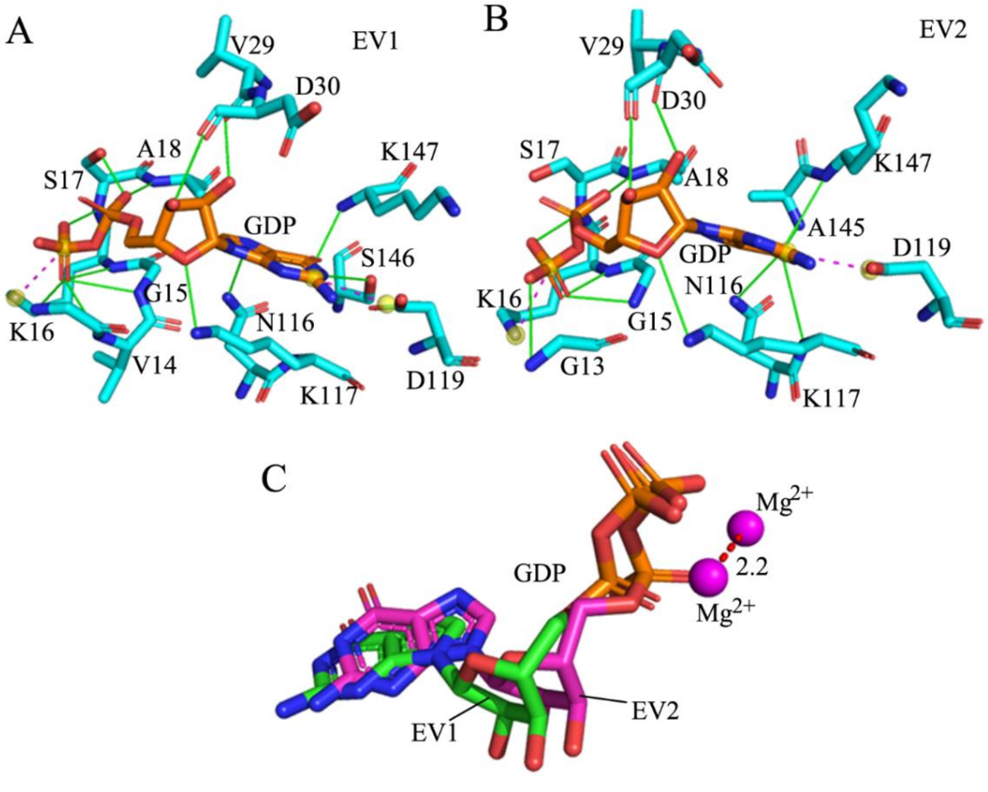


**Figure S1.** Interaction sites of GDP with NRAS and structural superimposition in the GDP-bound WT NRAS: (**A**) interaction sites of GDP with NRAS in the energetic state EV1, (**B**) interaction sites of GDP with NRAS in the energetic states EV2 and (**C**) structural superimposition of GDP and magnesium ions MG located at the EV1 and EV2.

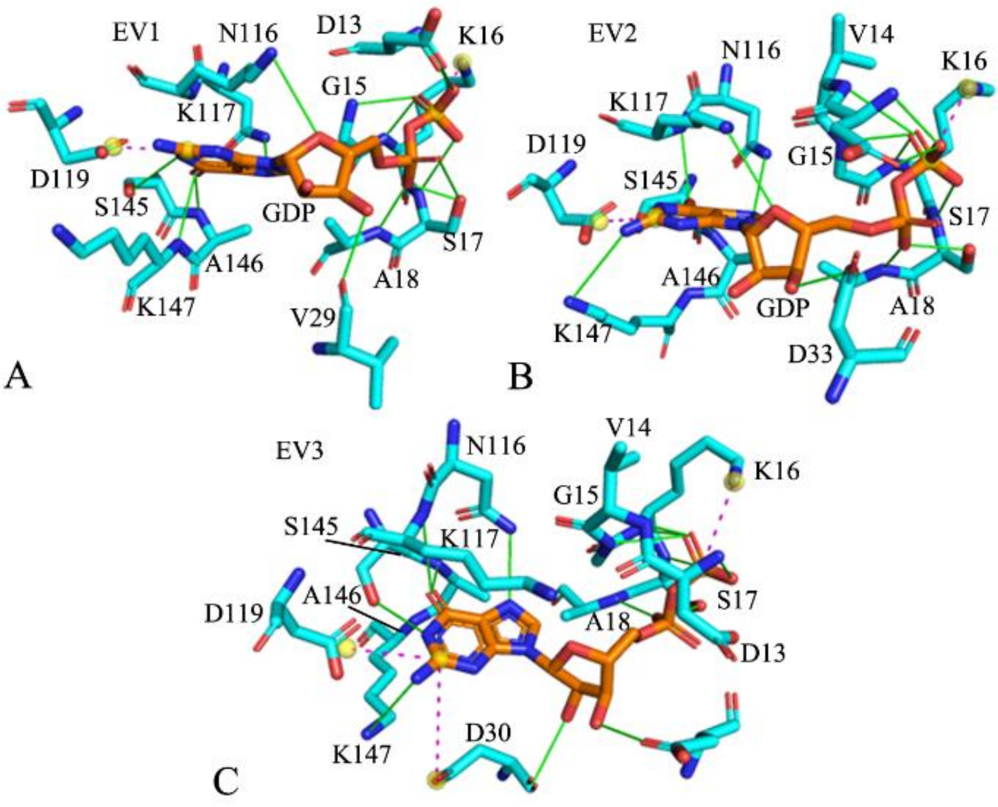


**Figure S2.** Interaction sites of GDP with NRAS in the GDP-bound G13D NRAS located at the different energetic states: (**A**) interaction sites of GDP with NRAS in the EV1, (**B**) interaction sites of GDP with NRAS in the EV2 and (**C**) interaction sites of GDP with NRAS in the EV3.

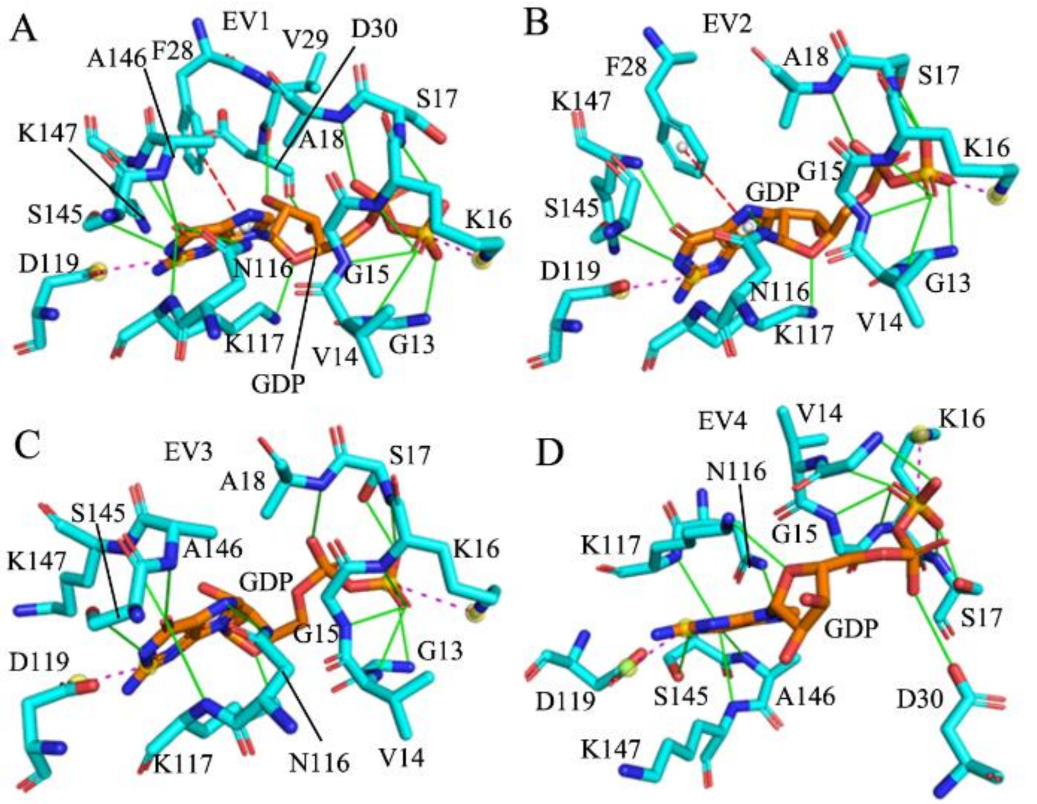


**Figure S3.** Interaction sites of GDP with NRAS in the GDP-bound Q61R NRAS located at the energetic states EV1-EV4: (**A**) interaction sites of GDP with NRAS in the EV1, (**B**) interaction sites of GDP with NRAS in the EV2, (**C**) interaction sites of GDP with NRAS in the EV3 and (**D**) interaction sites of GDP with NRAS in the EV4.

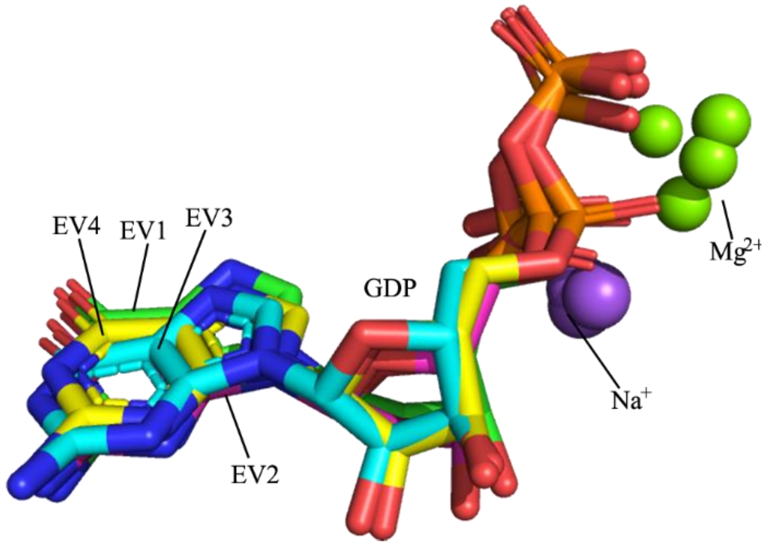


**Figure S4.** Superimposition of GDP and magnesium ions MG located at the energetic states EV1-EV4 of the GDP-bound Q61R NRAS.

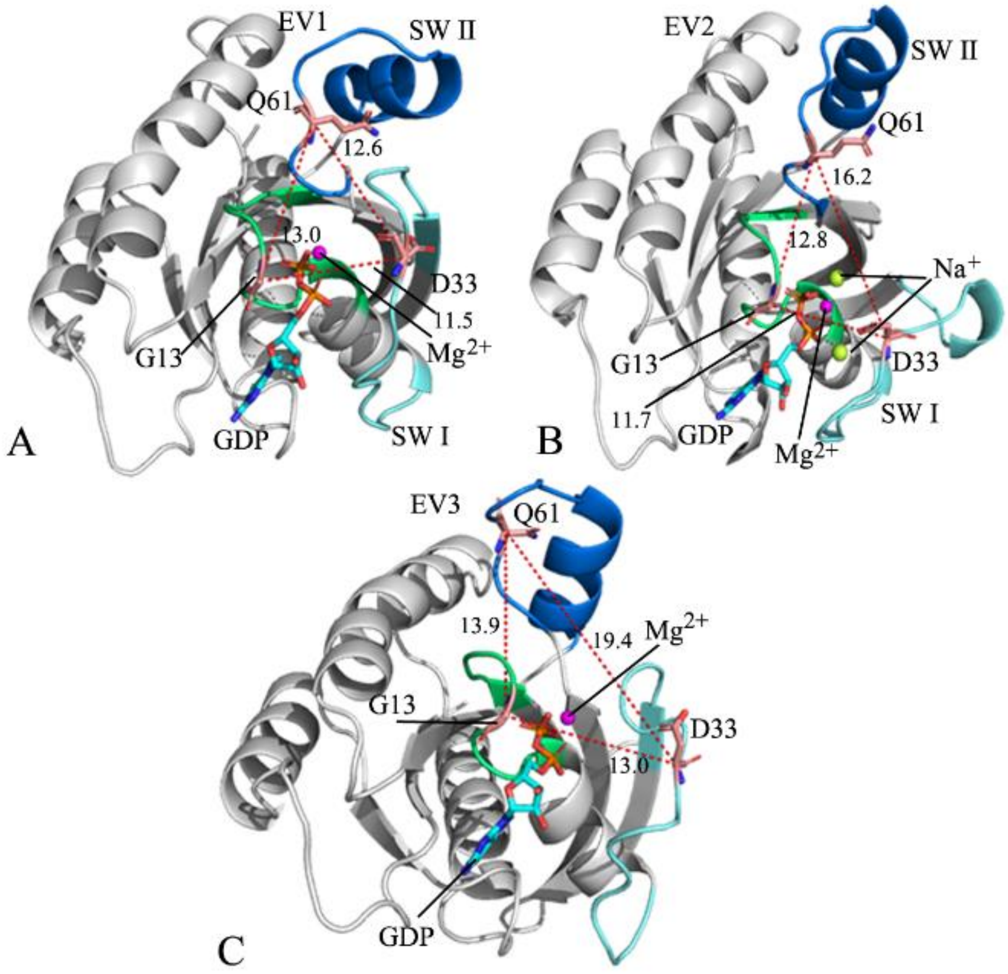


**Figure S5.** Representative structures of the GDP-bound C118S NRAS falling into the energy valleys EV1-EV3: (**A**) EV1, (**B**) EV2 and (**C**) EV3.

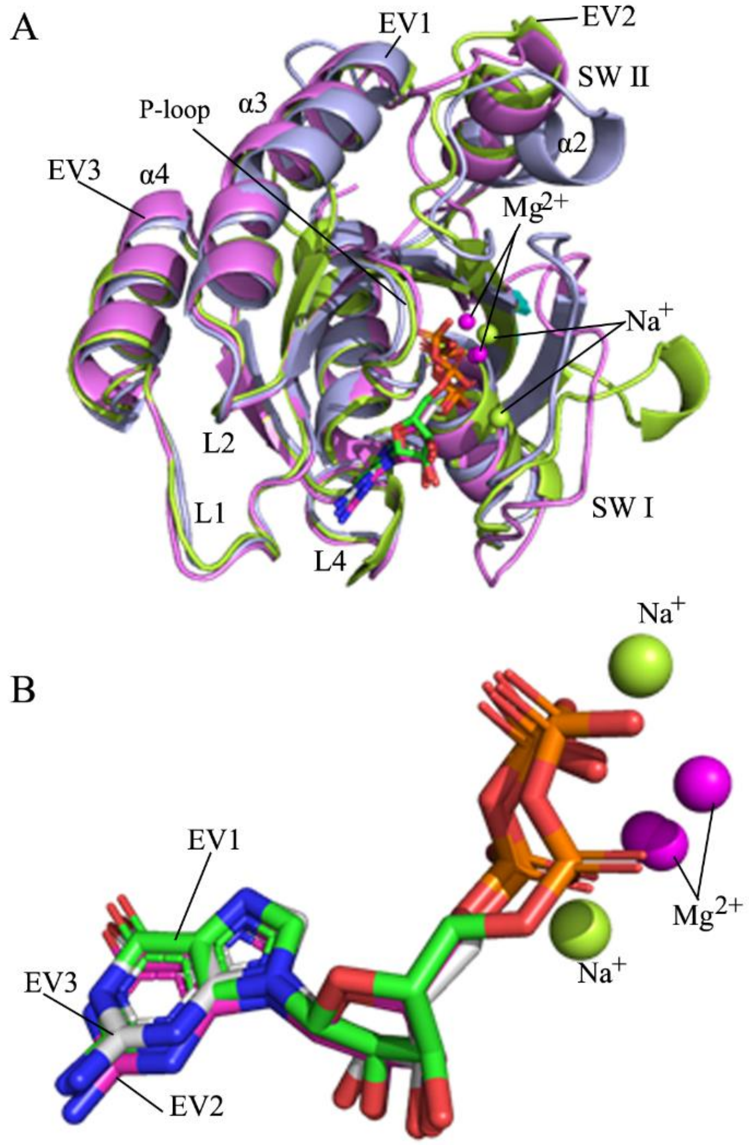


**Figure S6.** Superimposition of representative structures situated at the energy valleys EV1-EV3: (**A**) structural superimposition of the GDP-bound C118S NRAS and (**B**) structural superimposition of GDP and magnesium ions MG.

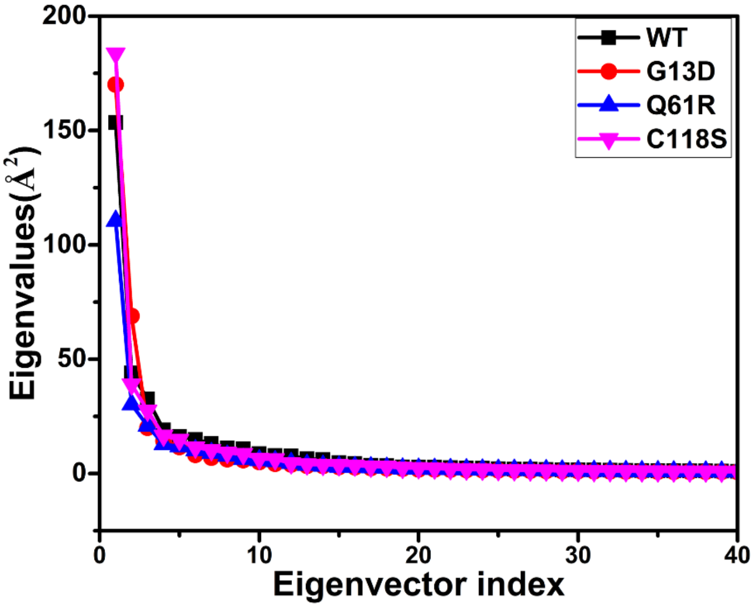


**Figure S7.** Function of eigenvalues VS eigenvector indexes from principal component analysis, which is used to describe motion intensity of NRAS.
